# Anisotropic PCL nanofibers embedded with nonlinear nanocrystals as strong generators of polarized second harmonic light and piezoelectric currents†

**DOI:** 10.1039/c9na00687g

**Published:** 2020-02-03

**Authors:** César R. Bernardo, Rosa M. F. Baptista, Etelvina de Matos Gomes, Paulo E. Lopes, Maria Manuela M. Raposo, Susana P. G. Costa, Michael S. Belsley

**Affiliations:** Univ. Minho, Centre of Physics Campus Gualtar 4710-057 Braga Portugal rosa_batista@fisica.uminho.pt; Univ. Minho, Inst. Polymers & Composites IPC Campus Azurém 4804-533 Guimarães Portugal; Univ. Minho, Centre of Chemistry Campus Gualtar 4710-057 Braga Portugal

## Abstract

Using the electrospinning technique nanofibers consisting of organic nonlinear optical 3-nitroaniline (3NA, C_6_H_6_N_2_O_2_) nanocrystals embedded in poly-*ε*-caprolactone (PCL) polymer, 3NA@PCL nanofibers, were produced. Polarimetry optical second harmonic generation and X-ray diffraction studies show that 3NA push–pull molecules crystallize inside the polymer fibers with a strong preferential orientation giving rise to an alignment of the molecular dipole moments along the nanofibers longitudinal axis. This alignment strongly enhances the second order nonlinear optical response of the fibers. Intense second harmonic generation emission was observed from a single nanofiber, corresponding to an effective second order susceptibility of 80 pm V^−1^, four times greater than the largest second order susceptibility tensor element (21 pm V^−1^) associated with a macroscopic 3NA crystal. Moreover, when subjected to a modest periodically applied force of 3 N, a piezoelectric current of 70 nA generated by a 4 cm^2^ electrospun nanofiber mat amounted to 122 nW cm^−2^ of instantaneous density power, sufficient to power a LCD display. The results show that the electrospinning technique is a powerful technique to fabricate organic functional materials with oriented nanocrystals made of highly polarizable molecules, embedded in a polymer matrix.

## Abstract 2

Intense second harmonic generation emission was observed from a single nanofiber, corresponding to an effective 2nd order susceptibility of 80 pm V^−1^, 4 times greater than the largest 2nd order susceptibility tensor element (21 pm V^−1^) for a macroscopic 3NA crystal.

## Graphical abstract

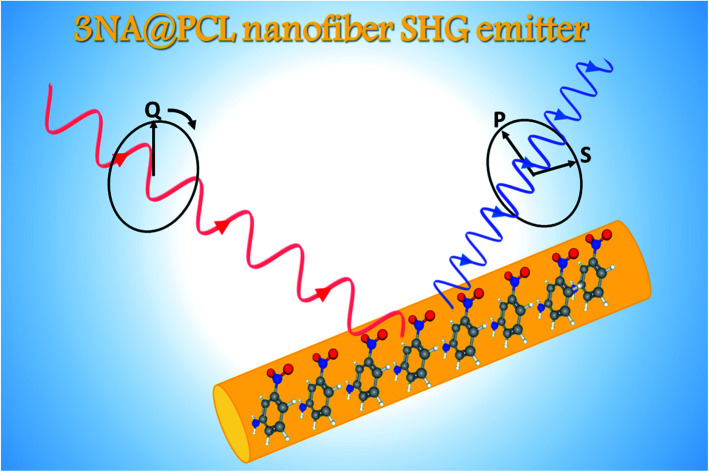

## Introduction

Low dimensional nanostructures in the form of fibers, wires, rods, belts, tubes, and rings have attracted attention due to their novel properties and potential applications as sensors, flexible electronic devices or nanogenerators of electrical energy. Compared to other methods of fabricating one-dimensional nanostructures, electrospinning is a simple and versatile technique capable of generating nanofibers from a variety of polymers with embedded organic or semi-organic nanocrystals forming hybrid composite materials.^1−3^ The technique has recently been employed to organize nonlinear optical (NLO) chromophores into subwavelength scale architectures with rationally designed functionalities.^4–8^

Molecular materials with highly efficient nonlinear optical (NLO) properties play an important role in modern technologies with applications in light frequency conversion and integrated optics.^9^

Nitroaniline derivative molecules are known for their large microscopic molecular hyperpolarizabilities and for those crystallizing into an acentric point group, a large second harmonic generation (SHG) response has been measured for most of them. The molecules have an admixture of zwitterionic D^+^–π–A– character in the ground state that reverses in the excited state. Assuming a two-level sum over-states model, this electronic structure gives origin to a strong first hyperpolarizability due to a change in the dipole moment orientation between the ground and excited state.^10^

*para*-Nitroaniline or 4-nitroaniline (pNA, C_6_H_6_N_2_O_2_), which serves as a model in molecular NLO exhibits very large molecular first order and second order polarizability.^11,12^ The molecule is formed by a benzene ring with a donor NH_2_ and acceptor NO_2_ groups in *para*-positions on the benzene ring.

*meta*-Nitroaniline or 3-nitroaniline (3NA, C_6_H_6_N_2_O_2_), is an isomeric molecule of pNA with the acceptor NO_2_ group in the position three of the benzene ring. Although their molecular dipole moments have similar magnitudes, respectively 51.46 D for pNA and 45.02 D for 3NA,^11^ they crystallize in different structures. While pNA crystals have a center of symmetry, 3NA crystallizes in the polar point group *mm*2.^13,14^ The crystal is biaxial with refractive indices *n*_1_ = 1.805, *n*_2_ = 1.715, *n*_3_ = 1.675 and the principal axis electro-optic constants are *r*_33_ = 16.7 pm V^−1^, *r*_23_ = 0.1 pm V^−1^, *r*_13_ = 7.4 pm V^−1^ at 632.8 nm, combining a low dielectric permittivity with a strong electro-optic behavior.^15,16^ 3NA crystals also display strong nonlinear optical effects due to its high second order susceptibility coefficients *d*_33_ = 21 pm V^−1^, *d*_32_ = 1.6 pm V^−1^*d*_31_ = 20 pm V^−1^.^17^ Furthermore, it exhibits piezoelectric coefficients which are similar in magnitude to those of the well-known nonlinear optical and piezoelectric lithium niobate, LiNbO_3_, crystal.^18^ 3NA was recently reported to display ferroelectric behavior.^3,19^

Good optical quality molecular organic crystals are more difficult to growth than inorganic crystals but their performance as excellent quadratic nonlinear crystals largely exceed that of inorganic ones. Growing nitroaniline derivative nanocrystals under the form of thin films or nanofibers has been recently explored with a view of developing them for applications in ultrafast optics.^20–22^

A strong nonlinear optical response was reported from electrospun fibers of 2-methyl-4-nitroaniline (MNA, C_7_H_8_N_2_O_2_) embedded in poly-l-lactic acid (PLLA) polymer. The observed intense second harmonic response resulted from a high degree of orientation of the MNA molecules inside the nanocrystal lattice originating a net dipolar moment along the nanofiber longitudinal axis.^3^ Moreover, it was demonstrated that by tuning the electrospinning parameters the second harmonic nonlinear response of MNA nanocrystals embedded into the nanofibers increased by an order of magnitude.^6^ This molecule is an engineered pNA molecule in which a methyl CH_3_ group has been substituted in the 2-position of the benzene ring to achieve crystalline noncentrosymmetry.^23^

Although 3NA molecules have been studied in depth both in solution and as macroscopic crystals, unlikely pNA^24–27^ and MNA its properties at nanoscale have not been addressed, in particular its SHG and piezoelectric behaviour when incorporated into polymer nanofibers.

In this work we report a strong polarized SHG response from a single electrospun nanofiber formed by well-oriented 3NA organic nanocrystals embedded in poly-*ε*-caprolactone (PCL) polymer. An effective second order susceptibility coefficient *d*^3NA@PCL^_eff_ = 80 pm V^−1^ has been measured in a nanofiber, which is four times larger than that reported for a macroscopic crystal. This is the first time 3NA high hyperpolarizable molecules have been integrated in functional nanofibers and their crystalline nonlinear optical response studied.

Also, upon applying a periodical force of 3 N to a 3NA@PCL nanofiber mat, a piezoelectric current of 70 nA was generated with a maximum output power density of 122 nW cm^−2^. This power is sufficient to operate a LCD display, making 3NA@PCL nanofibers mats suitable for use as flexible piezoelectric nanogenerators.

## Experimental

### Materials and electrospinning of nanofibers

Nanofibers were produced by a conventional electrospinning technique described previously.^3^ A 10% polymer solution formed by 0.5 g of 3NA and 0.5 g of poly-*ε*-caprolactone (PCL, *M*_w_ 80 000) on a 1 : 1 weight ratio were dissolved in 1 mL of dimethylformamide (DMF) and 4 mL of dichloromethane (DCM) solvents mixture. The chemicals were all purchased from Aldrich and used as received. The resulting clear and homogenous solution was stirred for several hours under ambient conditions prior to the electrospinning process. The precursor solution was loaded into a syringe with its needle connected to the anode of a high voltage power supply (Spellmann CZE2000). To produce the in-plane fibers the spinning voltage was set at 16 kV. The distance between anode and collector was 12 cm and precursor solution flow rate of 0.15 mL h^−1^ was controlled by a syringe pump with attached needle of 0.5 mm diameter. The fiber mat for piezoelectric measurements was collected on high purity aluminium foil which served as electrodes. For optical second harmonic generation measurements individual fibers were collected using a transparent glass substrate.

### Scanning electron microscopy (SEM)

The morphology, size and shape of 3NA@PCL nanofibers was studied using a Nova Nano SEM 200 Scanning Electron Microscope operated at 10 KV accelerating voltage.

### X-ray diffraction and Raman spectroscopy

Crystallinity and crystallographic orientation of 3NA nanocrystals inside the electrospun fibers was studied by X-ray diffraction. The diffraction pattern using *θ*–2*θ* scans was recorded between 10°and 60° on a Philips PW-1710 X-ray diffractometer with Cu-K_α_ radiation of wavelength 1.5406 Å. The lattice planes parallel to the substrate surface were determined from the reciprocal lattice vector of modulus (2/*λ*)sin *θ*, with *λ* the radiation wavelength and *θ* the Bragg angle. Raman spectroscopy was carried out on a LabRAM HR Evolution confocal Raman spectrometer (Horiba Scientific, France) using Horiba Scientific's Labspec 6 Spectroscopy Suite Software for instrument control, data acquisition and processing. The Raman spectra was obtained using a laser excitation with wavelength 532 nm, at 0.1% laser intensity, with 30 s acquisition time in a spectral range between 50–1750 cm^−1^.

### Optical absorption and emission

Optical absorption measurements on a 3NA solution were carried out using a Shimadzu UV/2501PC spectrophotometer, while photoluminescence spectra were collected using a FluoroMax-4 spectrofluorometer. For optical absorption measurements, a 1.0 × 10^−4^ M solution of 3NA was prepared in tetrahydrofuran (THF). The sample was measured in a quartz cuvette with 1 cm path length. Emission spectra from a 1.0 × 10^−4^ M solution of 3NA and from a 3NA@PCL nanofiber mat, were acquired using an excitation wavelength of 380 nm. The input and output slits were fixed to provide a spectral resolution of 5 nm.

### Optical second harmonic generation

Second harmonic generation (SHG) light efficiency of an individual 3NA@PCL fiber was measured using a polarimetry setup based on a mode-locked Ti:Sapphire laser (Coherent, Mira), a 100 fs temporal pulse width and a 76 MHz repetition rate. The polarization properties of SHG were measured to assess the orientation of 3NA crystals embedded within a nanofiber. Two different polarization curves were taken. For the q–p configuration, the analyzer was aligned along the direction that gives rise to the maximum SHG signal while a half-wave plate placed in a stepper motor controlled rotation stage was used to vary the incident field polarization. For the q–s configuration, a second half-wave plate behind the sample but before the analyzer was then rotated so that the detected polarization was perpendicular to that of the q–p configuration. To characterize the response of individual fibers the incident beam at 800 nm was focused using an Olympus 10× plan achromatic microscope objective to an estimated full-width at half maximum diameter of approximately 3 μm. A second 10× microscope objective collimated the generated second harmonic light which was subsequently detected by a cooled CCD mat (Andor Newton) after being spectrally resolved by a 0.3 monochromator (Andor Shamrock) with a 2400 lines per mm grating. The combination of the second wave plate and fixed analyzer orientation allows us avoid problems such as the variation of grating efficiency with polarization, ensuring that q–p and q–s curves have the same normalization.

### Piezoelectric current

The piezoelectric output voltage was measured across a 100 MΩ load resistance connected to a low pass filter followed by a low noise pre-amplifier (Research systems SR560) before being registered by a Digital Storage Oscilloscope (Agilent Technologies DS0-X-3012A). The nanofiber mat was submitted to periodic mechanical forces imposed by a vibration generator (Frederiksen SF2185) with a frequency of 3 Hz imposed by a signal generator (Hewlett Packard 33120A). The forces applied were measured by a calibrated force sensing resistor (FSR402, Interlink Electronics Sensor Technology). During the electrospinning process, the electrospun fibers were directly deposited on high purity aluminium foil, which served as electrodes. A mat with 4 cm^2^ area and 600 μm thickness was used. All the electric contacts are very stable. During the piezoelectric measurements there are no short circuit across the fiber mat, otherwise no output electric current would be measured. The sample mat is, previously to any measurements, checked for no short circuit between the electrodes.

## Results and discussion

### Fibers morphology, optical absorption and emission

**Fig. 1:**
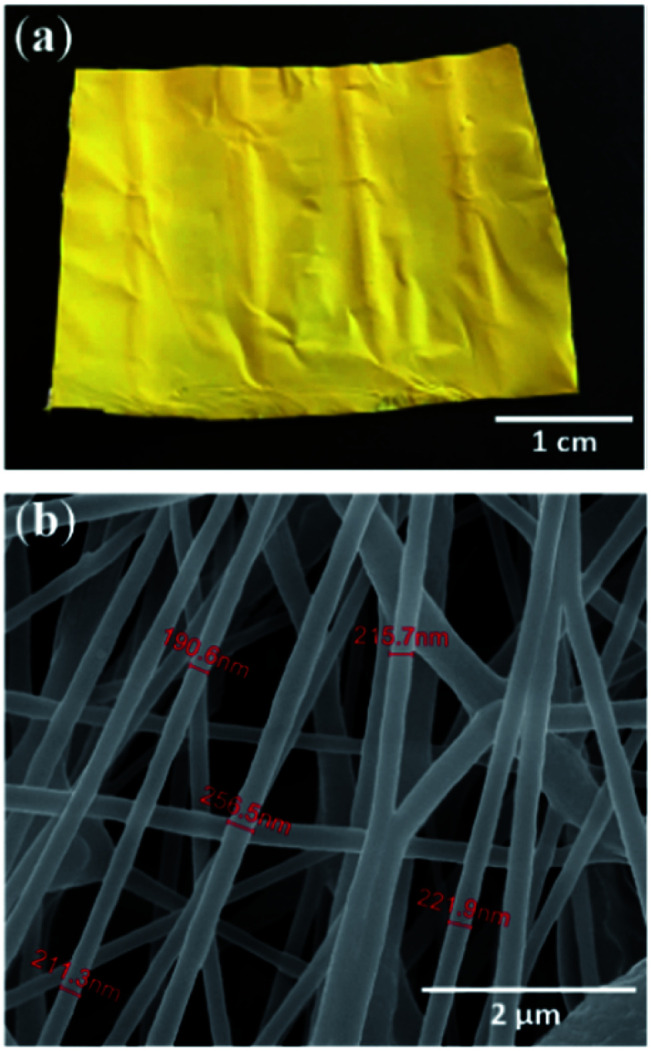
(a) 3NA@PCL electrospun fiber mat deposited on a substrate. (b) Corresponding SEM image.

The electrospun fibers are uniformly shaped, yellow colored with an average diameter of 230 nm forming a mat of continuous nanofibers, as indicated in Fig. 1(a) and (b) showing respectively the deposited fibers and the corresponding SEM observation. There are no beads and no 3NA crystals evident on the external fiber surfaces, all crystals are embedded within the fiber interior. To achieve these morphological characteristics, the electrospinning experimental conditions such as solution needle feeding rate, distance from the needle to the collector and voltage applied during the electrospinning process were carefully tuned.

**Fig. 2:**
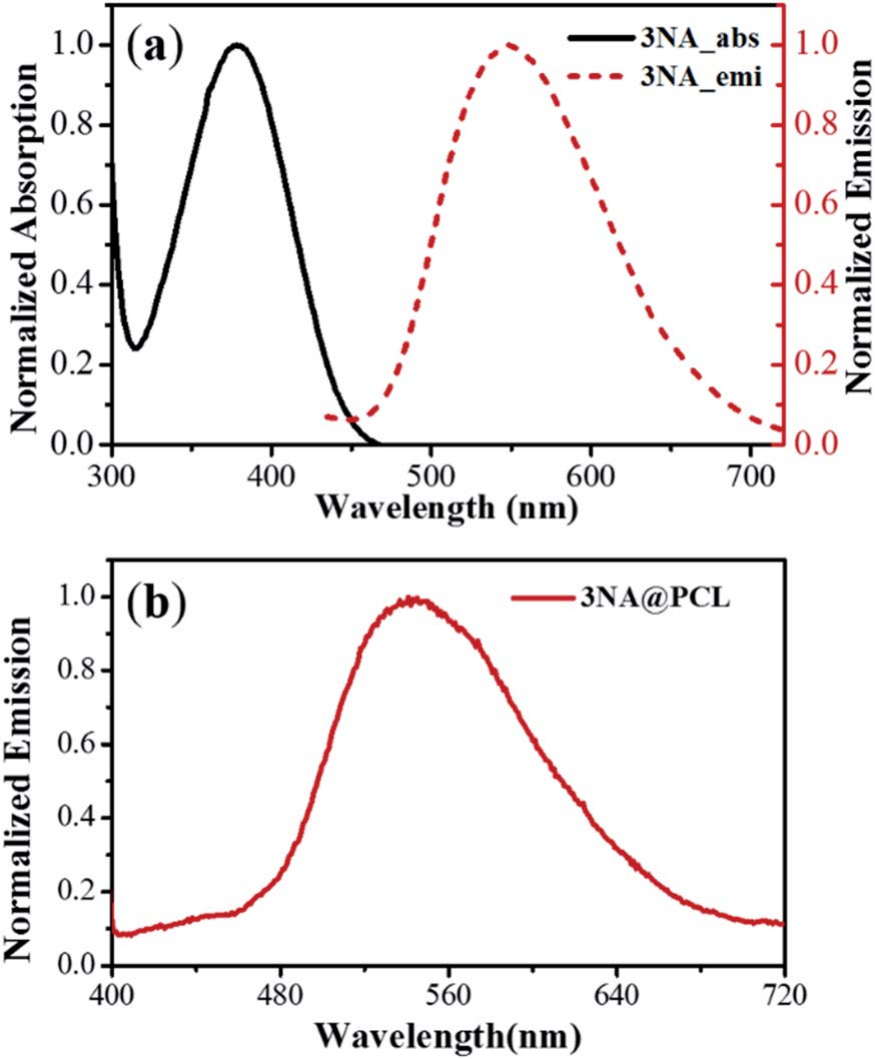
(a) Linear absorption and emission curves of 3NA in a THF solution. (b) Emission curve from a 3NA@PCL fiber mat.

The optical absorption and emission curves of 3NA molecules in a THF solution are shown in Fig. 2(a) and emission from nanocrystals embedded into electrospun fibers in Fig. 2(b). The 3NA@PCL fibers have a maximum emission at 550 nm similar to 3NA in solution and are transparent in the range of 450–700 nm as reported for bulk 3NA crystals.^17,18^

### X-ray diffraction and Raman spectroscopy

**Fig. 3:**
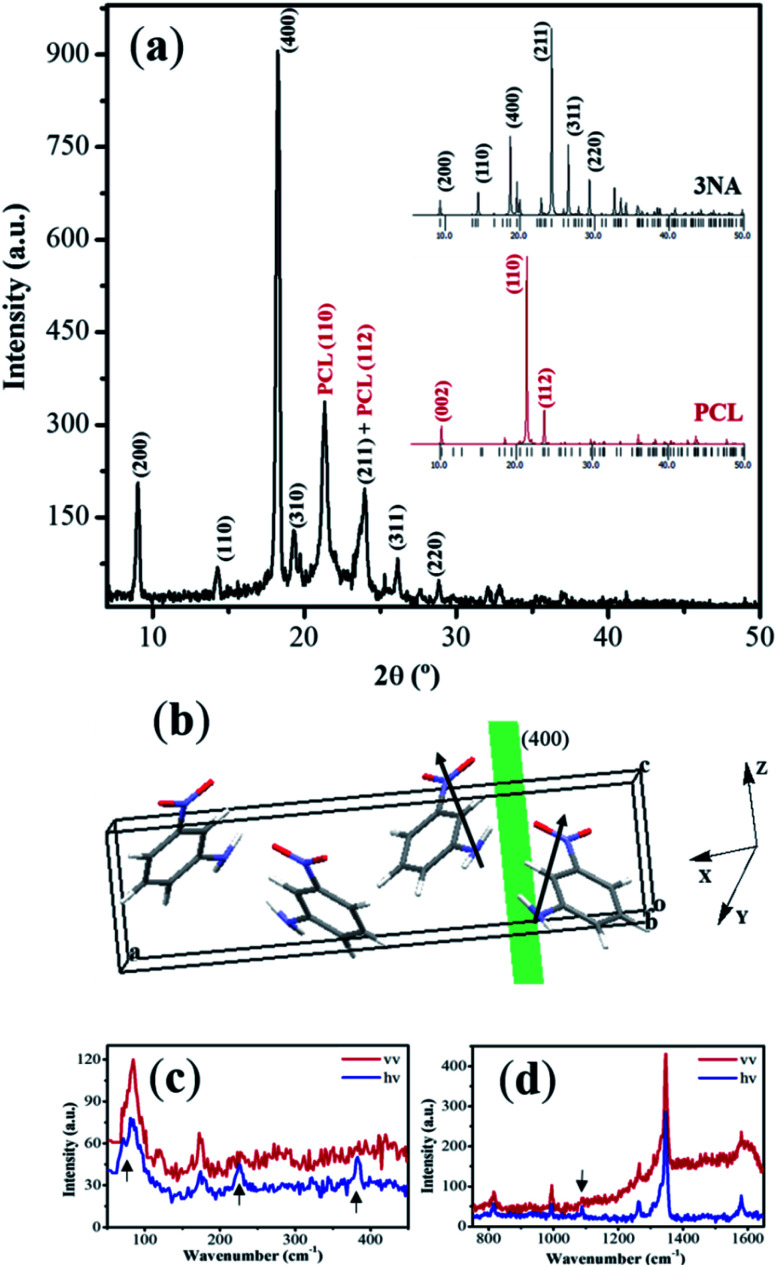
(a) Measured X-ray diffraction pattern of a 3NA@PCL nanofiber mat (the insets show the calculated powder patterns for crystalline 3NA and PCL polymer). (b) Unit cell content of 3NA showing the molecular dipoles (represented by arrows) adding to a net dipole parallel to (400). (c) and (d) Polarized Raman spectra of a 3NA@PCL nanofiber.

Bulk crystals of 3NA crystallize in the orthorhombic *Pca*2_1_ space group, point group *mm*2, with four molecules per unit cell.^13,28^ The molecules are almost planar and each consists of an aromatic benzene ring with the acceptor nitro (NO_2_) group in the *para*-position while the amino (NH_2_) donor group occupies the *ortho*-position originating a molecular dipole moment pointing from donor to acceptor groups. The crystalline structure of 3NA can be described as all-parallel polar layers perpendicular to [100] and interconnected by weak C–H⋯O bonds.^29^Fig. 3(a) is the X-ray diffraction powder pattern measured on a 3NA@PCL fiber mat and insets marked 3NA and PCL are those calculated for bulk 3NA crystals and PCL polymer, obtained using the program Mercury and published crystallographic information file.

The diffractograms show that for bulk 3NA the most intense Bragg reflection is (211) followed by (400) and (311) both with half intensity of (211), all the others reflections with much smaller intensities. For PCL there are only three intense Bragg reflections with (110) an order of magnitude more intense than the other two. However, the measured X-ray pattern of an electrospun nanofiber mat shows that the (400) Bragg reflection is the most intense and is followed by (200) reflection. Furthermore, (211) reflection is now roughly five times less intense than (400). This indicates that for 3NA@PCL electrospun mats there is a strong preferential orientation as 3NA molecules crystallize inside the fibers such that the crystallographic plane (400) aligns with the fiber mat plane. Fig. 3(b) shows the crystal unit cell content where 3NA molecules are arranged with their molecular dipole moment (represented by arrows) adding up to a net dipole moment parallel to (400) and pointing along the polar axis. This preferential orientation is induced by the strong electric field applied during the electrospinning process which tends to align the high molecular dipole moments within the crystal lattice, as reported before.^3^ We may therefore envision a 3NA@PCL electrospun fiber mat as a hybrid functional composite material formed by highly oriented 3NA nanocrystals embedded within a polymer matrix.

Polarized Raman spectroscopy indicates that some bands are absent, marked with an arrow on Fig. 3(c) and (d) in one of the spectra, consistent with the preferential nanocrystalline orientation inside the fibers as concluded from the X-ray powder data collected on 3NA@PCL and above described.

### Second harmonic generation

**Fig. 4:**
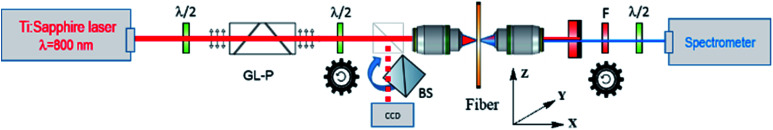
Polarimetry setup for optical second harmonic generation measurements built in our laboratory. The experiment is controlled through a LabVIEW program. In the figure: *λ*/2 – half wave-plate; GL-P – glan-laser calcite polarizer; F – short-pass filter; BS – beam splitter.

The highly oriented 3NA nanocrystalline arrangement inside the fibers should lead to a very anisotropic nonlinear optical response for light traveling with its wave vector perpendicular to the fiber longitudinal axis. To verify this hypothesis, the SHG efficiency of a single 3NA@PCL nanofiber was measured using a custom built polarimetry setup shown in Fig. 4.

The polarization properties of the generated second harmonic light (SHG) were measured to access the existence of preferred orientation for 3NA crystals embedded within the nanofibers. For crystals belonging to point group *mm*2 and taking into account the conditions of Kleinman symmetry,^30^ the second order optical polarizability components can be related to the incident fundamental electric field amplitudes by the matrix relation,^31^1
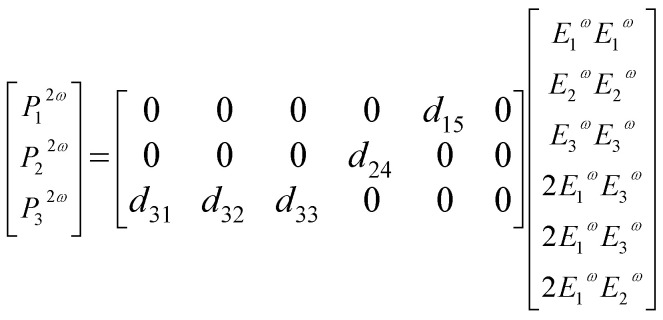
with *d*_15_ = *d*_31_ and *d*_24_ = *d*_32_. Here, *E*_1_^*ω*^, *E*_2_^*ω*^ and *E*_3_^*ω*^ are the electric field vector components of the fundamental optical field applied along *x*,*y*,*z* or 1,2,3 dielectric crystal axes, respectively. For an orthorhombic lattice the dielectric axes coincide with the crystallographic axes (see Fig. 3(b) and 4). When the fundamental light is incident along the *x* axis, the detected signal will vary with the incident field polarization angle *θ* according to the following relations,2
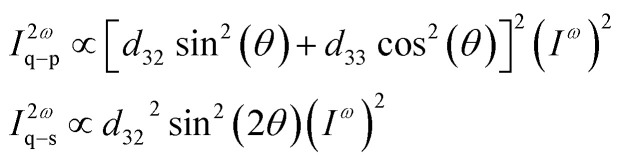


The polarimetry SHG measurements were performed on a single fiber and it is assumed that the light wave vector is, for most of the 3NA nanocrystals, along the dielectric *x* axis (see Fig. 4).

**Fig. 5:**
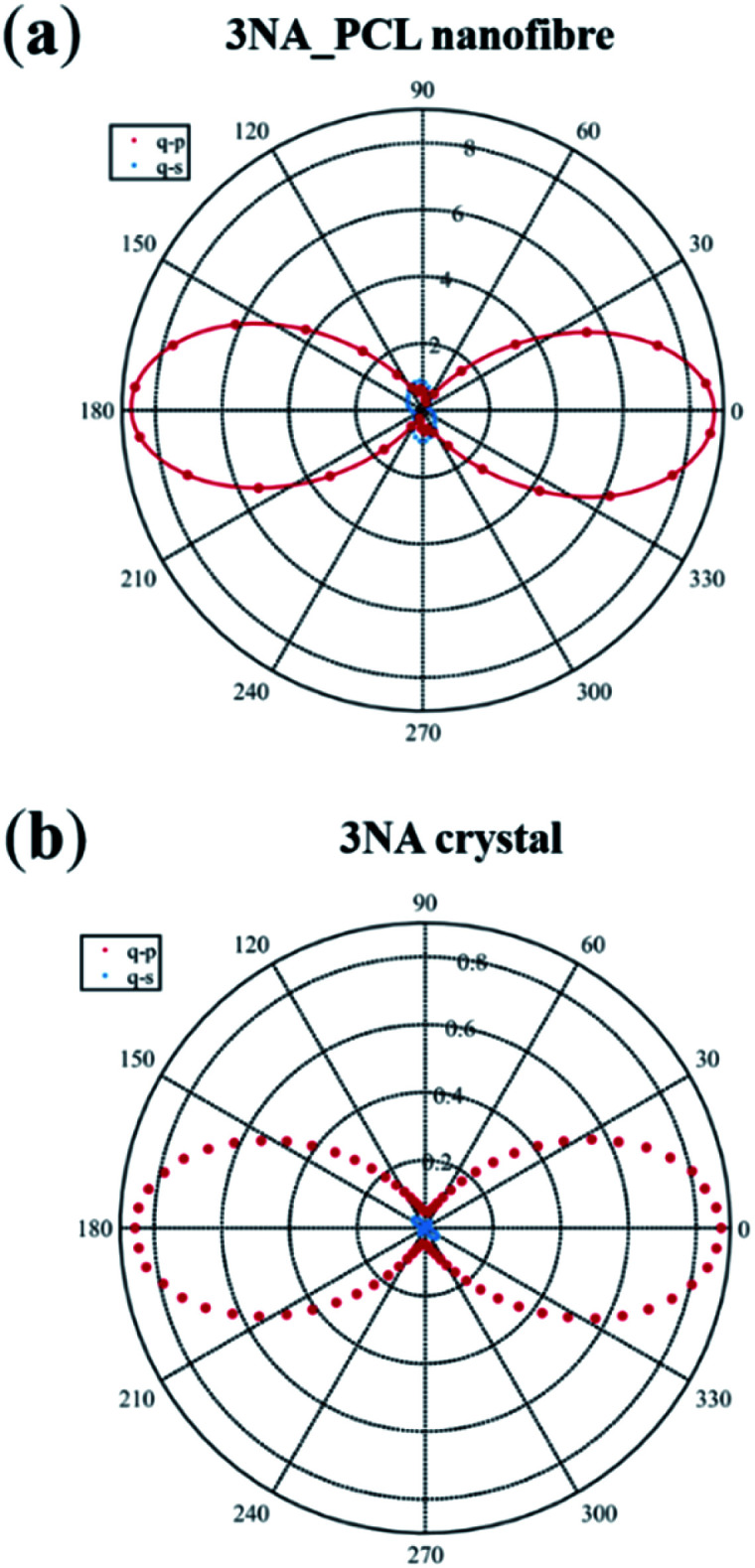
(a) Polar plot of SHG polarimetry data collected on a single 3NA@PCL electrospun nanofiber for q–p and q–s configurations. (b) Polar plot of SHG polarimetry data collected on a (100) 3NA crystal platelet for q–p and q–s configurations. The radial axis values are expressed in unit of counts. The maximum of intensity corresponds to the case where the polarization of incident and emitted light is parallel to each other and aligned with the fiber longitudinal axis.

The measured polar plots, Fig. 5 were acquired by integrating the signal over 0.25 seconds with an average incident energy of 6.5 pJ. Here, the maximum of intensity occurs when the polarization of the incident and emitted light are parallel to each other and aligned with the fiber longitudinal axis. For a 3NA@PCL fiber, the q–p polarimetry curve of Fig. 5(a) closely approximates a single cos^4^ *θ* pattern, confirming the strong preferred crystallographic orientation of 3NA nanocrystals embedded into the PCL polymer matrix. For a bulk 3NA crystal the second order polarizability tensor element *d*_33_ has a magnitude one order greater than *d*_32_,^10^ therefore in q–p configuration *d*_33_ is the main contributor to the measured intensity. For q–s configuration the measured intensity curve as shown in Fig. 5(b) is one order of magnitude smaller than q–p. Given the ratio of *d*_33_ to *d*_32_ one might expect that the q–s signal should be yet another order of magnitude smaller. The strong contrast in intensity between q–p and q–s curves indicates a strong degree of alignment of nanocrystals inside each fiber and reinforces the conclusion taken from the analysis of X-ray diffraction data.

For comparison we present the obtained polarimetry curves measured on a (100) oriented 3NA single crystal platelet with 0.45 mm thickness under the same excitation conditions and the incident beam normally incident on the platelet. The anisotropy of polarity curves is identical to that of 3NA nanocrystals embedded into the fibers, confirming the preferred orientation of the nanocrystals. Although the platelet thickness is three orders of magnitude higher than a typical nanofiber, remarkably the measured SHG output intensity of the platelet is lower than that of the nanofiber. This lower output from the bulk crystal reflects the phase mismatch in the SHG generated by the platelet. There is no phase mismatch for nanocrystals inside the fibers, as their size is much smaller than the coherence length of a 3NA crystal which is around 10 μm,^17^ which contributes to explain the nanofiber enhanced SHG response, together with the high alignement of the molecular dipoles along the fiber longitudinal axis.

The observed SHG enhancement displayed by electrospun fibers dopped with highly hyperpolarized nitroanilines derivative molecules crystallized in a polymer matrix, has been reported before MNA nanocrystals embedded in a PLLA polymer.^3^ In this work it is again demonstrated that the electrospinning technique is an efficient method for producing polymer doped nanofibers with a high degree of guest molecular polar orientation within the polymer and may be used to design a class of all-organic devices.

To determine the effective nonlinear susceptibility coefficient from a nanofiber, *d*^3NA@PCL^_eff_, the SHG response of the electrospun fiber was measured against an oriented beta barium borate (BBO) crystal of 1 mm thickness. This allows us to calibrate the collection efficiency of the set-up, type I phase matching occurs in BBO for an incident field at 800 nm that propagates at 29.2° relative to the optic axis with an effective second order nonlinear coefficient of *d*^BBO^_eff_ = 2.0 pm V^−1^.^32^ Using an 10× objective, the effective length over which the fundamental and second harmonic beam remains superimposed with the BBO crystal is limited by the 69 meridian walk-off angle of the extraordinarily polarized second harmonic wave to distance *l*^BBO^_S_ of approximately 250 μm. By comparison, temporal walk-off between the fundamental and second harmonic beams is negligible under our conditions. Wang and Weiner^33^ have developed a theoretical expression to estimate the efficiency of SHG by ultrashort pulses. Given the tight focus produced by the microscope objective their expression reduces to the form:3
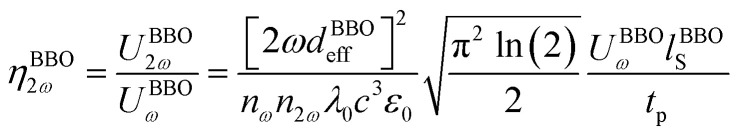
Here *U*_*ω*_ and *U*_2*ω*_ are the energies of the incident fundamental and generated second harmonic pulses while *t*_p_ is the FWHM pulse duration of the fundamental beam, roughly 100 femtoseconds. At phase matching the respective refractive indices *n*_*ω*_ and *n*_2*ω*_ are both equal to 1.660. In contrast, the submicron thickness of electrospun nanofibers allows one to use the standard phase matched plane wave result^33^ for SHG, which leads to a factor of *L*_3NA@PCL_^2^/*b*:4
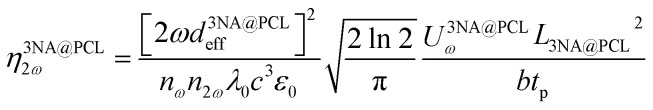
where *b* = 36 μm is the confocal length of the focused incident beam and *L*_3NA@PCL_ is the thickness of an individual fiber.

Taking the ratio between the two above expressions allows one to estimate the effective second order susceptibility coefficient of the electrospun fibers:5
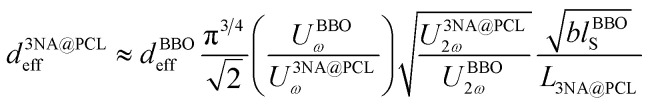
where we have neglected small corrections due to differences in the refractive indices. In our measurements the ratio of detected energies in q–p configuration was *U*_2*ω*_^3NA@PCL^/*U*^BBO^_2*ω*_ = 0.032 while the ratio of incident fundamental energies was *U*^3NA@PCL^_*ω*_/*U*^BBO^_*ω*_ = 10. However this latter factor should be corrected to take into account that an individual fiber with a diameter of 230 nm is significantly smaller than the estimated focused fundamental beam 1/*e*^2^ diameter of 3.3 μm, effectively reducing the ratio of incident energies to unity.

The estimated effective second order susceptibility coefficient for the q–p configuration is approximately *d*^3NA@PCL^_eff_ = 80 pm V^−1^. This is a factor of 4 greater than the largest tensor element *d*^3NA^_33_ = 21 pm V^−1^ for bulk crystalline 3NA.^17^ In conclusion, highly oriented 3NA nanocrystals inside each fiber behave as strong polarized nanoemitters of SHG light. This result indicates that it is feasible to produce a highly directional SHG emitter *via* the electrospinning technique.

### Piezoelectric response

Following the remarkable SHG response, an evaluation of the piezoelectric output voltage generated by a 3NA@PCL nanofiber mat when deformed by an external applied force was undertaken to access their suitability for potential integration into nanoenergy harvesting processes. The piezoelectric effect results from inter-conversion between mechanical and electrical stimulus inducing a charge redistribution and separation when a mechanical force is applied to a crystalline material. For any crystallographic point group the piezoelectric and SHG crystal properties are described by the same tensor,^31^ consequently the polarization *P*_*i*_ is related to the stress tensor *σ*_*j*_ by the matrix equation:6
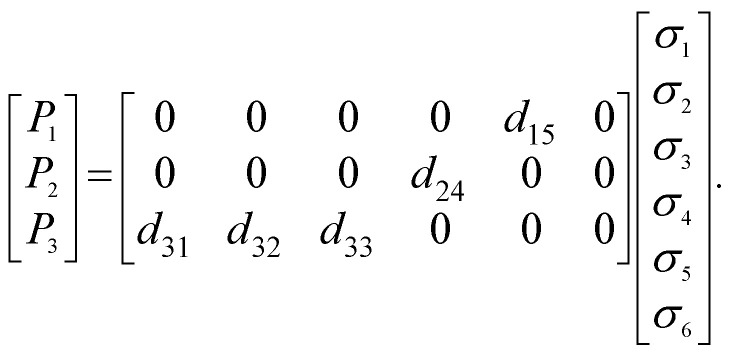


**Fig. 6:**
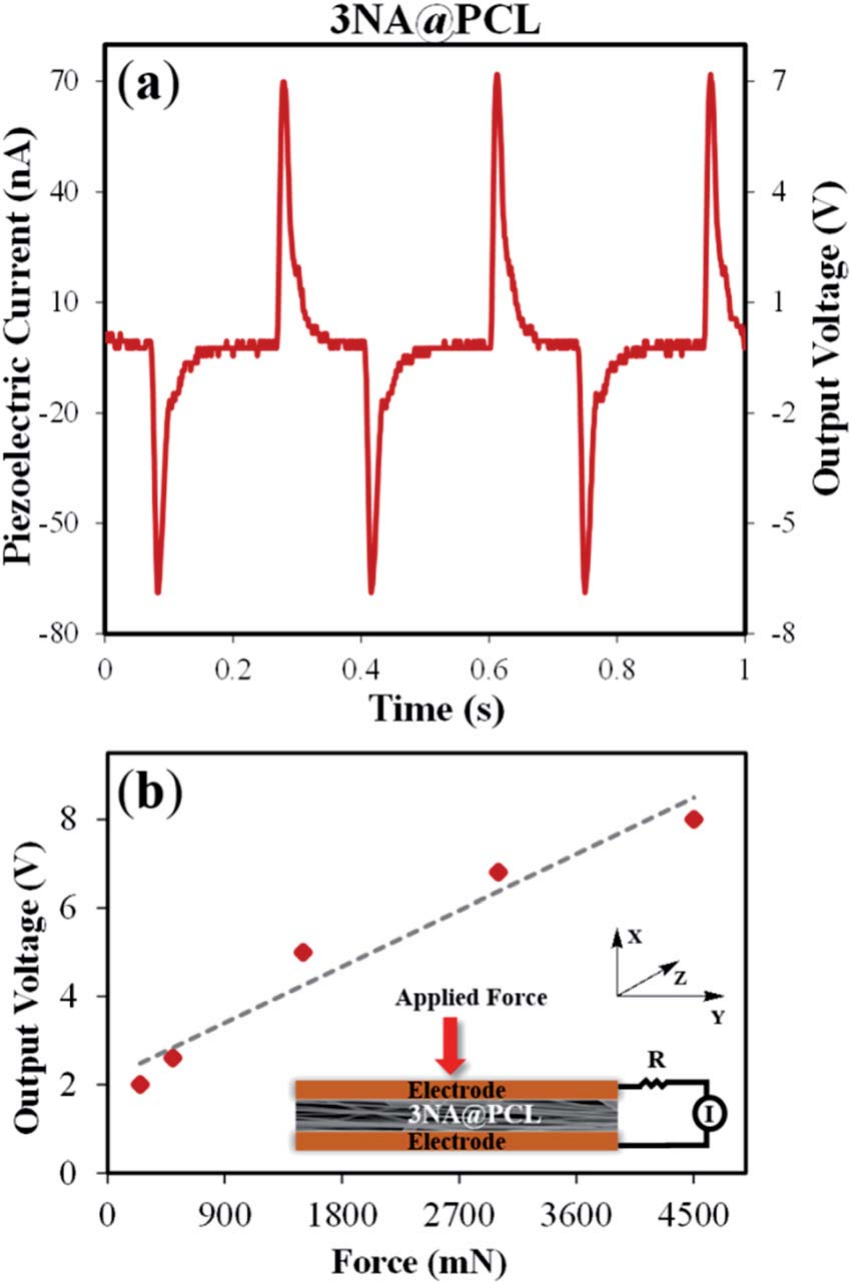
(a) Output voltage and current measured on a 3NA@PCL electrospun fiber mat. (b) Plot of output voltage *versus* applied force with a schematic piezoelectric setup of a 3NA@PCL fiber mat.

For bulk 3NA crystals the magnitude of the piezoelectric coefficients are *d*_33_ = 6.81 pCN^−1^, *d*_32_ = 2.55 pCN^−1^ and *d*_15_ = 30.79 pCN^−1^.^18^ The output voltage of 3NA@PCL electrospun fibers was measured by applying a periodical force perpendicularly to the fiber mat and measuring the polarization along the same direction, therefore along the *x* axis, as indicated in Fig. 6(b). According to the orientation of 3NA crystals embedded into the electrospun fibers above described and the piezoelectric tensor elements, eqn (6), the *d*_15_ shear coefficient is the main contributor to the piezoelectric response producing a polarization *P*_1_ = *d*_15_*σ*_5_, that is a force applied along *x* originates a polarization along the same direction due a shear stress *σ*_5_≡*σ*_13_.^31^ Consequently, the force applied along *x*(1) is transmitted across the crystal faces perpendicular to *z*(3) and therefore perpendicular to the molecular dipole moments, see Fig. 3(b).

As shown in Fig. 6(a), a voltage and current up to 7 V and 70 nA, respectively, were obtained when a force of 3 N was periodically applied. Fig. 6(b) is a plot of the output voltage as a function of the applied periodical force, showing a linear increase of the response with the magnitude of the force, as expected. 3NA fiber mats may be used as a power source for operating a LCD display: when pressing the electrospun fiber mat the letters “nA” are turned on (see video in ESI†).

For a 3NA@PCL fiber mat, an instantaneous density power of 122 nW cm^−2^ was achieved (calculated dividing the maximum electric power by the mat area). This power has a magnitude similar to that displayed by PVDF/PMLG [poly(vinylidene fluoride)/poly(γ-methyl l-glutamate)] composite fibers, six times greater than reported for nanofibers embedded with semi-organic ferroelectric DabcoHReO_4_ (1,4-diazabicyclo[2.2.2]octane perrhenate) and one order of magnitude smaller than obtained for inorganic ceramic ferroelectric BaTiO_3_ (barium titanate) and dipeptide diphenylalanine (Phe–Phe) nanofibers. This is indicated in Table 1.

**Table 1:** Piezoelectric output measurements from some electrospun nanofiber mats

Nanofibers	Power density (μW cm^−2^)	*V*_out_ (V)
3NA (present work)	0.12	7.0
P(VDF_TrFe)^34^	4.40	1.5
DabcoHReO_4_ (ref. 19)	0.02	0.12
Boc–Phe–Phe^35^	2.30	30.0
PVDF/PMLG^36^	0.13	0.19
BaTiO_3_ (ref. 37)	1.95	4.0

## Conclusions

We have fabricated hybrid electrospun nanofibers with nanometer thickness and lengths of several centimeters containing highly oriented nanocrystals of 3-nitroaniline, a nonlinear optical molecule with elevated hyperpolarizability, embedded in poly-*ε*-caprolactone polymer. The estimated effective second order susceptibility measured on a single nanofiber can reach values as high as *d*^3NA@PCL^_eff_ = 80 pm V^−1^, a factor of four times greater than the largest tensor element *d*^3NA^_33_ = 21 pm V^−1^ associated with macroscopic 3NA crystals. Therefore 3NA nanocrystals inside each fiber behave as enhanced polarized nanoemitters of SHG light.

Our results also demonstrate that by embedding 3NA molecules inside polymer fibers one is able to fabricate a macroscopy mat of crystalline hybrid functional nanofibers with strong piezoelectric response achieving an instantaneous power density of 122 nW cm^−2^. In summary, by embedding appropriately chosen organic molecules with large individual high hyperpolarizabilities into a suitable polymer matrix, the electrospinning technique can faster crystallization with strong preferential orientation within each fiber. Single nanofibers can generate enhanced and strong polarized second harmonic light, whereas nanofiber mat efficiently convert modest applied forces into piezoelectric currents.

## Conflicts of interest

The authors declare that they have no known competing financial interests or personal relationships that could have appeared to influence the work reported in this article.

## Supplementary Material

NA-002-C9NA00687G-s001
